# The Functional Characterization of GCaMP3.0 Variants Specifically Targeted to Subcellular Domains

**DOI:** 10.3390/ijms23126593

**Published:** 2022-06-13

**Authors:** Annika Kempmann, Thomas Gensch, Andreas Offenhäusser, Irina Tihaa, Vanessa Maybeck, Sabine Balfanz, Arnd Baumann

**Affiliations:** 1 Institute of Biological Information Processing, IBI-1, Research Center Jülich, 52428 Jülich, Germany; annika.kempmann@gmail.com (A.K.); t.gensch@fz-juelich.de (T.G.); s.balfanz@fz-juelich.de (S.B.); 2 Institute of Biological Information Processing, IBI-3, Research Center Jülich, 52428 Jülich, Germany; a.offenhaeusser@fz-juelich.de (A.O.); i.tihaa@gmail.com (I.T.); v.maybeck@fz-juelich.de (V.M.)

**Keywords:** Adeno-associated virus, cellular signaling, genetically encoded Ca^2+^ indicators, histamine receptors, optogenetic tools, primary cortical neurons, second messengers

## Abstract

Calcium (Ca^2+^) ions play a pivotal role in physiology and cellular signaling. The intracellular Ca^2+^ concentration ([Ca^2+^]_i_) is about three orders of magnitude lower than the extracellular concentration, resulting in a steep transmembrane concentration gradient. Thus, the spatial and the temporal dynamics of [Ca^2+^]_i_ are ideally suited to modulate Ca^2+^-mediated cellular responses to external signals. A variety of highly sophisticated methods have been developed to gain insight into cellular Ca^2+^ dynamics. In addition to electrophysiological measurements and the application of synthetic dyes that change their fluorescent properties upon interaction with Ca^2+^, the introduction and the ongoing development of genetically encoded Ca^2+^ indicators (GECI) opened a new era to study Ca^2+^-driven processes in living cells and organisms. Here, we have focused on one well-established GECI, i.e., GCaMP3.0. We have systematically modified the protein with sequence motifs, allowing localization of the sensor in the nucleus, in the mitochondrial matrix, at the mitochondrial outer membrane, and at the plasma membrane. The individual variants and a cytosolic version of GCaMP3.0 were overexpressed and purified from *E. coli* cells to study their biophysical properties in solution. All versions were examined to monitor Ca^2+^ signaling in stably transfected cell lines and in primary cortical neurons transduced with recombinant Adeno-associated viruses (rAAV). In this comparative study, we provide evidence for a robust approach to reliably trace Ca^2+^ signals at the (sub)-cellular level with pronounced temporal resolution.

## 1. Introduction

External signals evoke specific cellular responses by activating signal transduction processes that often cause transient changes of intracellular second messenger concentrations. Evidence has accumulated that a timely and spatially restricted modulation rather than a global change of second messenger concentrations, e.g., cyclic adenosine monophosphate (cAMP), cyclic guanosine monophosphate (cGMP), or Ca^2+^, is pivotal to cellular signaling [1,2,3,4,5,6,7,8,9].

Fine-tuning intracellular Ca^2+^ concentrations ([Ca^2+^]_i_) is physiologically very important to controlling muscle contraction, neurotransmission, as well as the activation of gene transcription [10,11,12,13]. Under resting conditions, the free cellular [Ca^2+^]_i_ is exquisitely clamped to a low value (≤1 µM). This is achieved by efficient transport mechanisms extruding Ca^2+^ ions from the cytosol to the extracellular side or to internal stores such as the endoplasmic reticulum or mitochondria [14,15,16]. In addition to transporters and Ca^2+^ pumps, Ca^2+^ homeostasis is maintained by Ca^2+^-binding proteins such as calmodulin, calbindin, or calreticulin in the cytosol [17,18]. These Ca^2+^ chelators assist in clamping [Ca^2+^]_i_ to low values since sustained increased [Ca^2+^]_i_ may have detrimental effects causing cell damage or even cell death [19].

In the course of studying cellular signaling at the molecular level, there was ongoing demand to monitor the transient and the local changes in [Ca^2+^]_i_. This interest triggered the development of a variety of strategies. The introduction of Ca^2+^-sensitive fluorescent dyes, such as Fura-2 or Fluo-4 [20,21], was a large achievement and allowed non-invasive registering and following of Ca^2+^ signals in living cells and tissue [22,23]. However, once having crossed the plasma membrane these dyes distribute rather equally within the cell and even enter subcellular compartments. Therefore, it is difficult to register local Ca^2+^ transients with synthetic fluorescent dyes. Another important issue is that Ca^2+^-sensing fluorescent dyes allow imaging of [Ca^2+^]_i_ only for a couple of hours after loading because cells continuously extrude or degrade them. These problems were overcome with the introduction of genetically-encoded Ca^2+^ indicators (GECI) [24,25,26]. Two main groups of sensors have been developed: Förster resonance energy transfer (FRET)-based sensors harbor calmodulin or troponin C as Ca^2+^ detectors that are fused to a pair of fluorescent proteins with overlapping emission and absorption spectra, the second group comprise a single fluorescent protein fused to a Ca^2+^ detector (e.g., GCaMP, RCaMP) (for reviews see [27,28,29,30]). Until today, new versions of both groups were developed to enable better temporal resolution and imaging in living organisms [31,32]. Members of both groups of sensors were frequently applied at the cellular level as well as in transgenic organisms and facilitated Ca^2+^ measurements under physiological conditions [33,34,35,36,37]. Since GECI are ideally suited for additional genetic engineering, some sensors have been modified to target the protein to subcellular domains allowing locally confined Ca^2+^ measurements [38,39,40,41,42,43].

We decided to employ the Ca^2+^ sensor GCaMP3.0 [34] for a systematic modification and functional application approach. In addition to the cytosolic variant (GCaMP3.0cyto), we engineered different versions that localize in the nucleus (GCaMP3.0nuc), in the mitochondrial matrix (GCaMP3.0mito), at the mitochondrial outer membrane (GCaMP3.0mom), and at the plasma membrane (GCaMP3.0pm). Biophysical properties of each recombinant protein were characterized in aqueous solution on purified samples obtained after overexpressing the proteins in *E. coli*. In addition, the functionality and the specificity of all variants were examined in cell lines stably transfected with these constructs and in primary cultures of cortical neurons transduced with recombinant Adeno-associated viruses (rAAV). We provide evidence that the targeting sequences (a) have a minute influence on the biophysical parameters of purified GCaMP3.0 versions, (b) allow precise and specific localization of the sensors to designated domains in cells and primary neurons, and (c) support measuring Ca^2+^ signals evoked by different stimuli with high temporal and spatial resolution.

## 2. Results

### 2.1. Design and Cellular Distribution of Targeted GCaMP3.0 Variants

We used a GCaMP3.0-encoding recombinant plasmid for modification and construction of specifically targeted sensor variants. All constructs were modified at the 5′ end with a unique restriction site for cloning purposes and a Kozak consensus sequence (CCACC) [44] preceding the ATG codon of the open reading frame for efficient translation. The original untargeted construct of GCaMP3.0 is mainly localized in the cytosol (GCaMP3.0cyto; Figure S1). For localization at the plasma membrane, the nucleotide sequence was extended at the 5′ end by a sequence motif encoding the N-terminal 21 amino acid residues of neuromodulin (Growth Associated Protein 43, GAP43) [45,46]. This motif contains two neighboring cysteine residues (C_3_/C_4_) that can be palmitoylated and then target the protein to the plasma membrane. To support sufficient flexibility for calmodulin (CaM) to bind to the M13 peptide preceding the circular permutated eGFP in GCaMP3.0, we inserted a spacer of 28 amino acid residues between the GAP43 motif and the initiating methionine of the M13 peptide. The final construct was called GCaMP3.0pm. Notably, when the membrane-targeting sequence was fused to the 5′-end of the construct without a spacer, the protein was not targeted to the membrane. Similarly, adding the targeting sequence to the 3′-end of the construct, i.e., fusing it to the calmodulin domain of GCaMP3.0, again resulted in mis-localization and impaired functionality of the GECI.

Various processes of cellular signaling and physiology are controlled by free [Ca^2+^]_i_. Mitochondria are known to play a key role in controlling [Ca^2+^]_i_ due to efficient sequestration and release mechanisms that have been intensively studied, e.g., with a variety of synthetic or genetically encoded Ca^2+^ indicators ([47] Here, for localization in the mitochondrial matrix, the GCaMP3.0 sequence was extended at the 5′ end by a segment encoding the first 36 amino acid residues of cytochrome C oxidase subunit VIII (Cox8) [48,49]. The final construct was called GCaMP3.0mito.

Positioning GCaMP3.0 at the outer mitochondrial membrane was achieved by adding 33 amino acid residues including the transmembrane anchor sequence of the mitochondrial transport protein TOM20 [50] to the N-terminal end of GCaMP3.0. The final construct was called GCaMP3.0mom.

To achieve transport of GCaMP3.0 into the nucleus, we extended the N-terminus of the sensor by seven amino acid residues originating from the SV40 large T-antigen (PKKKRKV) [51]. The final construct was called GCaMP3.0nuc.

**Figure 1:**
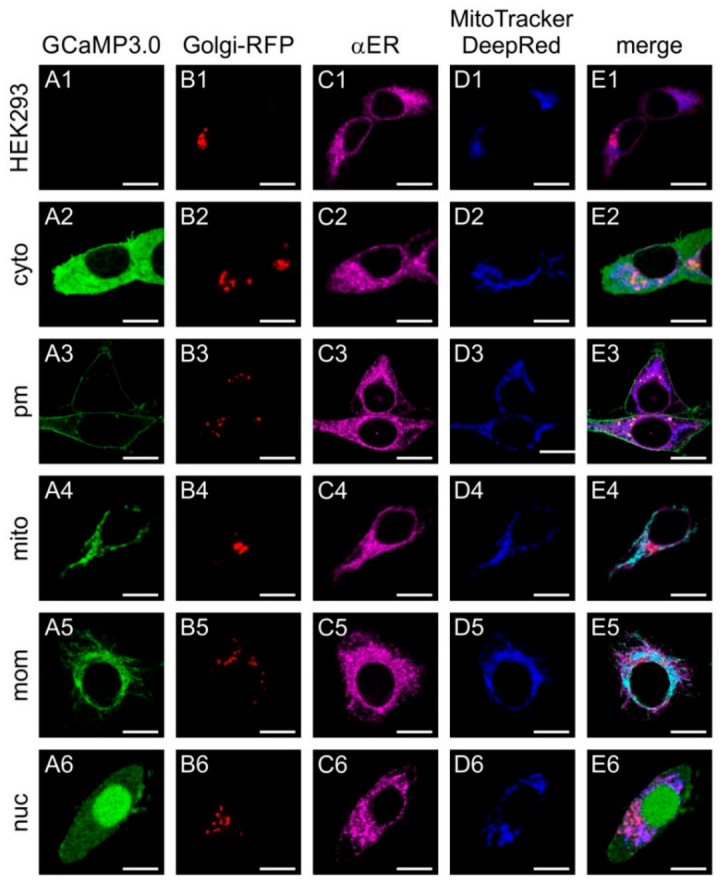
**Sub-cellular distribution of transiently expressed GCaMP3.0 variants**. Human embryonic kidney cells (HEK293) were either non-transfected with a GCaMP3.0 encoding construct (**A1**–**E1**) or with GCaMP3.0cyto (**A2**–**E2**), GCaMP3.0pm (**A3**–**E3**), GCaMP3.0mito (**A4**–**E4**), GCaMP3.0mom (**A5**–**E5**), or GCaMP3.0nuc (**A6**–**E6**). Expression of GCaMP3.0 variants was detected by their green fluorescence (**A2**–**A6**). For identificationof the Golgi apparatus in cells, they were transfected with Golgi-RFP (**B1**–**B6**). The endoplasmic reticulum was stained with primary rabbit anti-calnexin antibodies (dilution 1:100) and secondary donkey anti-rabbit-A594 (dilution 1:1000) antibodies (**C1**–**C6**). Labeling of mitochondria was performed with MitoTracker DeepRed FM (**D1**–**D6**). Fluorescent images were obtained by confocal imaging of the samples. Composite images are depicted in (**E1**–**E6**). The scale bar denotes 8 µm.

The constructs were cloned into eukaryotic expression vectors and used for transient transfection of human embryonic kidney cells (HEK293) to monitor their subcellular distribution (Figure 1). In order to visualize the Golgi apparatus, cells were co-transfected with a recombinant plasmid encoding Golgi-RFP. Localization of GCaMP3.0 variants was monitored via their intrinsic green fluorescence. The endoplasmic reticulum (ER) was labeled with a specific antibody (α-calnexin) and a fluorescently labeled secondary antibody (dk-α-rbA594). Mitochondria were stained with MitoTracker Deep Red FM.

As expected, non-transfected cells did not emit fluorescent signals of GCaMP3.0, but the Golgi apparatus, ER, and mitochondria were specifically stained (Figure 1(A1–E1)). Cells transfected with the cytosolic variant (GCaMP3.0cyto) showed a rather even distribution of the protein mainly in the cytosol (Figure 1(A2)). All other markers showed a similar pattern (Figure 1(B2–E2)) as observed for the negative control (Figure 1(B1–E1)). The variant harboring the GAP43 motif localized to the outer borders of the cells (Figure 1(A3)) and did not overlap with any of the other structures (Figure 1(B3–E3)). Localization of GCaMP3.0 containing the Cox8 sequence showed a non-homogeneous distribution (Figure 1(A4)) that overlapped with the MitoTracker staining (Figure 1(D4,E4)). The construct containing the TOM20 motif (Figure 1(A5)) also showed a good overlap with the MitoTracker signal (Figure 1(D5,E5)). Finally, the GCaMP3.0nuc version showed preferential localization in the cell’s nucleus with only small amounts of protein being present in the cytosol (Figure 1(A6,E6)).

Having proven that the modified sensors were targeted to their pre-destined loci in HEK293 cells, we set out to examine whether the biophysical properties of the modified proteins were preserved or eventually altered compared to GCaMP3.0cyto.

### 2.2. Expression, Purification, and Characterization of GCaMP3.0 Variants

The constructs were cloned into pRSET A vector (Invitrogen/Thermo Fisher Scientific, Darmstadt, Germany) that allows expression of the protein in *E. coli* and simultaneously adds a hexa-histidine-tag (His_6_) to the N-terminus of the protein. The GCaMP3.0 variants were purified via Ni-NTA affinity chromatography and then applied to in vitro spectroscopic characterization.

**Figure 2:**
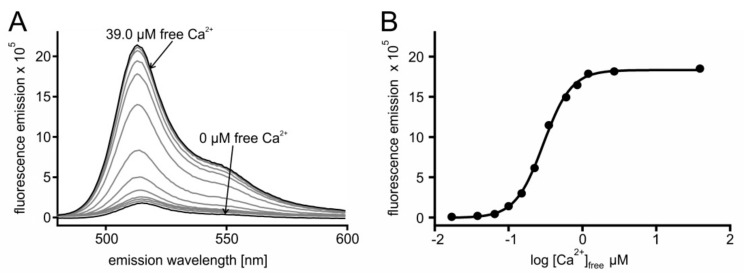
**In vitro characterization of GCaMP3.0nuc.** The construct was cloned into pRSET A vector, over-expressed in *E. coli* (BL21(DE3)LysS cells and affinity purified via Ni-NTA agarose. Purified GCaMP3.0nuc protein (2 µM final concentration) was diluted in incubation buffer (30 mM MOPS, 100 mM KCl, pH 7.2) adjusted to the desired [Ca^2+^]_free_ with CaEGTA (10 mM)/K_2_EGTA (10 mM). (**A**) For each [Ca^2+^]_free_ the fluorescence emission spectrum of GCaMP3.0nuc was registered upon excitation at 470 nm. Spectra obtained at 0 µM and 39.0 µM [Ca^2+^]_free_ are indicated with arrows. Maximal fluorescence intensity was detected at 510 nm. (**B**) Fluorescence intensity at 510 nm was plotted against log [Ca^2+^]_free_. The Ca^2+^-concentration–response curve was calculated with GraphPad Prism (5.04). Data represent the values from a typical experiment. Experiments were independently repeated at least four times for each construct (see Table 1).

In a series of experiments, the Ca^2+^ affinity of the sensors was determined. Briefly, 2 µM protein were incubated with increasing concentrations of Ca^2+^ ([Ca^2+^]_free_). For each [Ca^2+^] the sample was excited at 470 nm and the fluorescence emission spectrum was registered. A representative example is depicted in Figure 2, showing results of GCaMP3.0nuc and a corresponding absorption spectrum is shown in Figure S1.

From the spectra (Figure 2A) the maximal fluorescence at 510 nm was extracted and plotted against [Ca^2+^]_free_. Fitting of the data resulted in the concentration–response curve displayed in Figure 2B. The fit resulted in an EC_50_ of 297 nM for GCaMP3.0nuc. Measurements for all other GCaMP3.0 variants followed the same regime. Calculation of mean EC_50_ ± SD are summarized in Table 1. The affinities of all constructs were statistically different up to 1.3-fold (One Way ANOVA test, *p* = 0.06). Additionally, the sensors dynamic ranges, i.e., the ratio of (fluorescence (F) F_max_/F_min_) were different up to 1.2-fold (Table 1).

**Figure 3:**
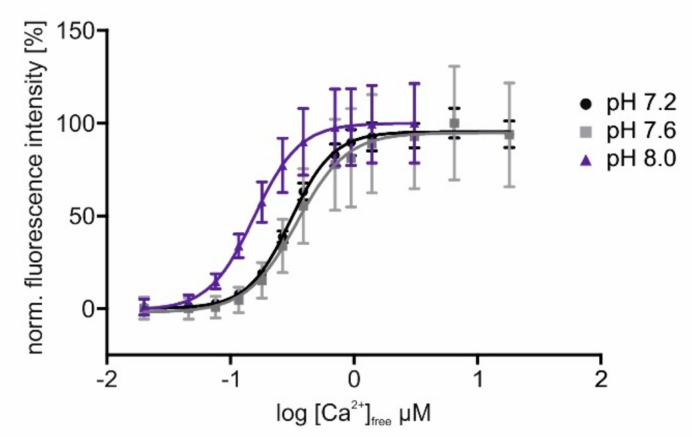
**In vitro characterization of GCaMP3.0mito at different pH values.** The GCaMP3.0mito construct was designed for localizing to the mitochondrial matrix. For in vitro characterization of the protein, the recombinant was cloned into pRSET A vector, over-expressed in *E. coli* (BL21(DE3)LysS cells and affinity purified via Ni-NTA agarose. Purified GCaMP3.0mito protein (2 µM final concentration) was diluted in an incubation buffer (30 mM MOPS, 100 mM KCl at pH 7.2 (black), pH 7.6 (gray), or pH 8.0 (purple)) and adjusted to the desired [Ca^2+^]_free_ with CaEGTA (10 mM)/K_2_EGTA (10 mM). For each [Ca^2+^]_free_, the fluorescence emission spectrum was registered upon excitation at 470 nm. Fluorescence intensities at 510 nm were normalized to the highest fluorescence intensity (=100%) obtained at the different pH values. Mean values from five independent experiments were plotted against log [Ca^2+^]_free_. The Ca^2+^-concentration–response curves were calculated with GraphPad Prism (5.04).

These results show that the introduced modifications did not change the sensors fluorescent and Ca^2+^-binding properties substantially. Since we aimed to position sensors in cellular compartments, e.g., the mitochondrial matrix, differing in pH from the cytosol, we measured the pH dependence of sensor responses to increasing [Ca^2+^]_free_. In Figure 3, the delineated concentration–response curves of Ca^2+^ titrations at three different pH values are depicted. For better comparison, fluorescence values were normalized to the highest intensity (=100%) measured in each sample. The data show that at a higher pH, as it is present in mitochondria [52], the sensor’s response saturates already at lower [Ca^2+^]_free_. Measurements at pH 7.2 or 7.6 have almost no or only minor effects on the sensor’s response profile. The changed values for EC_50_ and the dynamic range of GCaMP3.0mito at a higher pH are considerably below 1 µM [Ca^2+^], and they will not prevent the detection of physiologically relevant Ca^2+^ signals in mitochondria [53].

### 2.3. Functional Measurements of Ca^2+^ Dynamics in HEK293 Cells

We established HEK293 cell lines, each constitutively expressing one of the different sensors. We used these cell lines to examine the homogeneity and expression level of each sensor variant. In all cell lines, we found GCaMP3.0 localization as predicted by the targeting sequence. Line scan analyses of the subcellular distribution of GCaMP3.0 proteins are summarized in Figure S2.

**Figure 4:**
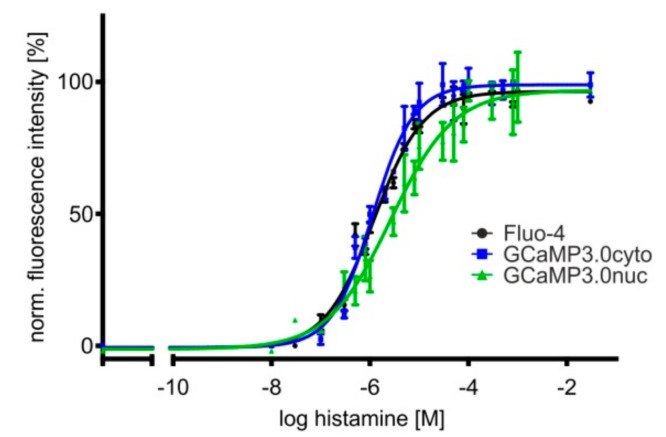
**Concentration–response curves of histamine-induced Ca^2+^ signals in HEK293 cell lines.** Cell lines were established constitutively expressing differently targeted GCaMP3.0 variants. Here, GCaMP3.0cyto and GCaMP3.0nuc expressing cells were compared with parental HEK293 cells loaded with the Ca^2+^-sensitive dye Fluo-4 for monitoring histamine-induced Ca^2+^ signals. Cells were seeded into 96 well dishes at a density of 20,000 cells/well. A change in [Ca^2+^]_i_ was registered as a change in fluorescence intensity (at 510 nm). Fluorescence values were normalized to the highest fluorescence intensity (=100%) obtained at the highest histamine concentration. Each data point (four-fold determination (±SD)) was plotted against the corresponding histamine concentration. Experiments were repeated independently at least three times. The concentration–response curves were calculated with GraphPad Prism (5.04).

In a previous study we reported that a particular clone of HEK293 cells endogenously expressed histamine receptors [54]. One of these GTP-binding-protein coupled receptors (GPCR), i.e., hH1, leads to PLC-mediated IP_3_ production followed by Ca^2+^ release from internal stores upon histamine binding. The same lineage of HEK293 cells was used to establish the cell lines expressing GCaMP3.0 variants. Therefore, we employed these cells to monitor Ca^2+^ responses upon application of a histamine concentration series. In addition to cell lines expressing GCaMP3.0cyto and GCaMP3.0nuc, control measurements were performed in non-transfected cells loaded with Fluo-4. Concentration–response curves are depicted in Figure 4.

The EC_50_ values obtained from fitting the data (at least three independent experiments with four-fold determination for each data point) are very similar with 1.2 ± 0.1 µM (Fluo-4), 1.1 ± 0.1 µM (GCaMP3.0cyto), and 2.7 ± 1.3 µM (GCaMP3.0nuc), demonstrating that differently targeted sensors are well suited for cell-based biosensor assays and can substitute for the application of Ca^2+^ sensitive dyes.

**Figure 5:**
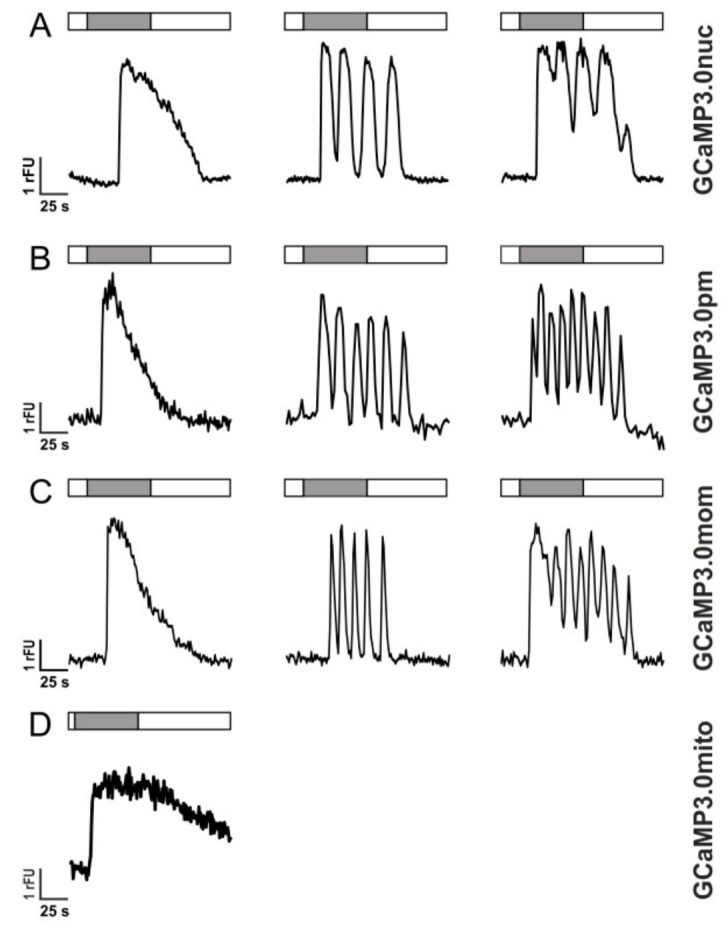
**Histamine-induced Ca^2+^ dynamics in HEK293 cells registered with GCaMP3.0 variants** HEK293 cells were stably transfected with GCaMP3.0 variants: (**A**) GCaMP3.0nuc, (**B**) GCaMP3.0pm, (**C**) GCaMP3.0mom, and (**D**) GCaMP3.0mito. Cells were superfused with extracellular solution (ES: 120 mM NaCl, 5 mM KCl, 2 mM MgCl_2_, 2 mM CaCl_2_, 10 mM HEPES, 10 mM glucose, pH 7.4; white bar) or with ES containing 10 µM histamine (gray bar). Ca^2+^-dependent fluorescence signals in individual cells were registered. Three types of signals were observed: (a) single transients (left row), oscillations (middle row), and oscillations on elevated basal fluorescence (right row). Except for GCaMP3.0mito (**D**) all other variants did not differ in detecting and tracing the cellular responses.

Activation of GPCRs, however, does not always result in single transient Ca^2+^ elevations but may also lead to Ca^2+^ oscillations most likely due to receptor de- and re-sensitization phenomena [54,55]. In Figure 5, responses of individual cells are depicted that were superfused with 10 µM histamine for 60 s. Three types of Ca^2+^-dependent fluorescent signals were registered: single transients (left panel), oscillations (middle panel), and oscillations on elevated [Ca^2+^] (right panel).

The variants GCaMP3.0nuc, GCaMP3.0pm, and GCaMP3.0mom registered these intracellular [Ca^2+^] signals similarly well as the established GCaMP3.0cyto sensor or non-transfected cells loaded with Fluo-4. The mitochondrial construct, GCaMP3.0mito, showed a completely different response profile. Upon histamine application, the fluorescence signal increased, remained at an elevated value and then slowly returned to the basal level after histamine application was stopped (Figure 5D). The proper orientation and localization of GCaMP3.0mito was further examined by uncoupling the mitochondrial respiratory chain with Carbonyl cyanide-p-trifluoromethoxyphenylhydrazone (FCCP).

**Figure 6:**
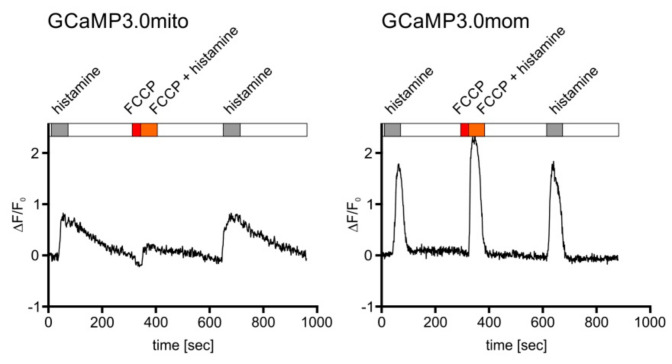
**Mitochondrial targeted GCaMP3.0 versions respond differently, yet specifically, to Ca^2+^ signals.** Cells constitutively expressing either the GCaMP3.0mito or GCaMP3.0mom version of the sensor were superfused with extracellular solution (ES: 120 mM NaCl, 5 mM KCl, 2 mM MgCl_2_, 2 mM CaCl_2_, 10 mM HEPES, 10 mM glucose, pH 7.4; white bar), with ES containing 10 µM histamine (gray bar), ES containing 10 µM FCCP (red bar), or ES containing 10 µM histamine and 10 µM FCCP (orange bar). Ca^2+^-dependent fluorescence signals in individual cells were registered. Application of FCCP and FCCP/histamine (red and orange bar) resulted in a strong reduction of the fluorescence signals. After wash-out of FCCP and subsequent stimulation with histamine, the fluorescence signal recovered and reached the initial value and shape. In cells constitutively expressing the GCaMP3.0mom variant, targeted to the mitochondrial outer membrane, application of FCCP did not result in a reduction of the Ca^2+^-dependent fluorescence compared to stimulation with histamine alone. For comparison, the change in fluorescence intensity normalized to basal fluorescence intensity (∆F/F_0_) is plotted against time ([s]).

This compound leads to depolarization of the mitochondrial membrane potential and causes a reduced influx of Ca^2+^ ions [56] upon histamine stimulation. A representative experiment is depicted in Figure 6. Treating cells expressing GCaMP3.0mito with FCCP resulted in lower fluorescence signals compared to cells before application or after wash-out of FCCP. Using the same experimental set-up for cells expressing GCaMP3.0mom (Figure 6, right panel) did not cause a decrease in fluorescence when cells were treated with FCCP. This result further supports the notion that GCaMP3.0mito is properly targeted to the mitochondrial matrix and suited to register inner-mitochondrial Ca^2+^ signaling. On the contrary, GCaMP3.0mom is localized at the outer mitochondrial membrane facing the cytosol and therefore reports the cytosolic [Ca^2+^].

### 2.4. Dynamics of Ca^2+^ Signals Registered with Mitochondrial GCaMP3.0 Variants and Fura-2

**Figure 7:**
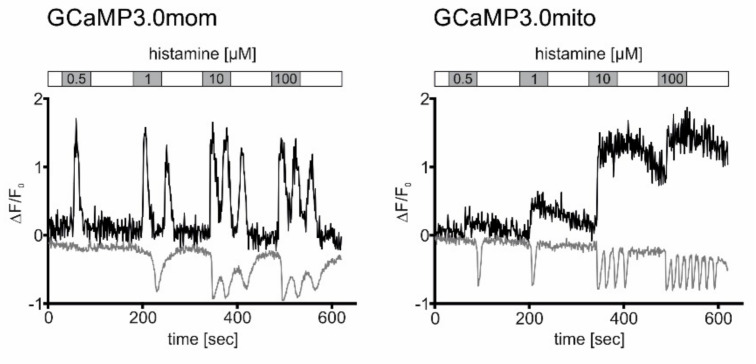
**Registration of histamine-evoked Ca^2+^ signals in cells expressing mitochondria-targeted GCaMP3.0 versions and loaded with Fura-2.** Cells constitutively expressing GCaMP3.0mom or GCaMP3.0mito were loaded with the Ca^2+^ sensitive dye Fura-2. Samples were superfused with extracellular solution (ES: 120 mM NaCl, 5 mM KCl, 2 mM MgCl_2_, 2 mM CaCl_2_, 10 mM HEPES, 10 mM glucose, pH 7.4; white bar) containing increasing histamine concentrations (0.5–100 µM; grey bars) for 60 s. Between stimulations, cells were superfused with histamine-free ES for 90 s. Ca^2+^-dependent fluorescence signals in individual cells were registered. Two-photon excitation (920 nm) was used to register Ca^2+^-dependent fluorescence emission from GCaMP3.0mom or GCaMP3.0mito during the stimulation protocol with histamine (black traces). Thereafter, the same cell was excited at (760 nm) to monitor Fura-2 responses using the same stimulation regime (grey traces). The change in fluorescence (∆F = F-F_0_) was normalized against the basal fluorescence (F_0_). Normalized values (∆F/F_0_) were plotted against the time (s). Binding of Ca^2+^ ions to GCaMP3.0 sensors causes an increase in fluorescence emission. Binding of Ca^2+^ ions to Fura-2 causes a decrease in fluorescence emission.

We extended our comparative analyses and measured Ca^2+^ signals in cells constitutively expressing mitochondrially-targeted GCaMP3.0 variants that were additionally loaded with the Ca^2+^-sensitive dye Fura-2. Both GCaMP3.0 and Fura-2 have emission maxima at 510 nm [20]. Their absorption spectra and consequently their two-photon excitation wavelengths, however, are different (GCaMP3.0 = 920 nm; Fura-2 = 760 nm). Fura-2 has two absorption bands with maxima at 380 nm (Ca^2+^-free form) and 340 nm (Ca^2+^-bound form) [20]. With our two-photon fluorescence microscope setup, only the Ca^2+^-free form of Fura-2 can be excited (760 nm). Therefore, intracellular elevation of [Ca^2+^] will lead to a decrease of fluorescence, opposite to increasing GCaMP3.0 signals. Similar to the previous experiments, Fura-2 loaded cells expressing either GCaMP3.0mom or GCaMP3.0mito were exposed to increasing concentrations of histamine (0.5 µM; 1 µM; 10 µM; 100 µM) for 60 s intermitted by washing steps with histamine-free extracellular solution for 90 s. The application of different histamine concentrations was performed twice. During the first experiment, excitation at 960 nm was used to record GCaMP3.0mom and GCaMP3.0mito signals. During the second application with excitation at 760 nm, Fura-2 signals were registered. Representative experiments are shown in Figure 7. Stimulation of cells with histamine caused changes of Ca^2+^-dependent fluorescence signals of both the GCaMP3.0 variants and Fura-2. The signals, however, differed with respect to their shape and their duration.

In cells expressing GCaMP3.0mom, a transient increase in Ca^2+^-dependent sensor fluorescence was detected with 0.5 µM histamine. Application of higher histamine concentrations caused oscillatory Ca^2+^-dependent signals. Binding of Ca^2+^ ions to Fura-2 causes a decrease of the fluorescence signal, when excited at 760 nm (see Figure 7). With Fura-2 a transient signal was registered at 1 µM histamine, followed by oscillations when higher ligand concentrations were applied.

In cells expressing GCaMP3.0mito, the shape of the signals differed clearly from those obtained with GCaMP3.0mom. Long-lasting transient signals were registered with the sensor located in the mitochondrial matrix. For small histamine concentrations (0.5 µM; 1 µM), fluorescence signals almost returned to base line values during the washing step (see Figure 7). At higher histamine concentrations, fluorescence intensity of the sensor increased and did not return to the base line value during the washing step. In contrast to GCaMP3.0mito fluorescence, transient Fura-2 signals were obtained at 0.5 and 1 µM histamine concentrations. Fluorescence completely returned to base line values during the washing steps. Higher histamine concentrations (10 µM; 100 µM) evoked oscillating Ca^2+^-dependent Fura-2 signals that returned to base line values once ligand-free extracellular solution was superfused onto the sample.

Since Fura-2 is homogeneously distributed in the cell, it primarily allows detection of cytosolic Ca^2+^-signals. Located at the outer membrane of mitochondria, the GCaMP3.0mom sensor registers changing [Ca^2+^]_i_ in the cytosol as well. Thus, it was not surprising that both Fura-2 and GCaMP3.0mom were able to monitor the oscillatory Ca^2+^ responses initiated by histamine and caused by Ca^2+^ release from intracellular stores. In contrast, GCaMP3.0mito located within mitochondria responded with transient yet increasing Ca^2+^ signals, most likely reflecting influx of Ca^2+^ ions into the organelle serving as an alternative route in cellular Ca^2+^-homeostasis and nicely demonstrates the potential of targeted Ca^2+^-sensors.

### 2.5. Measurements of Local Ca^2+^ Signals in Primary Cortical Neurons

**Figure 8:**
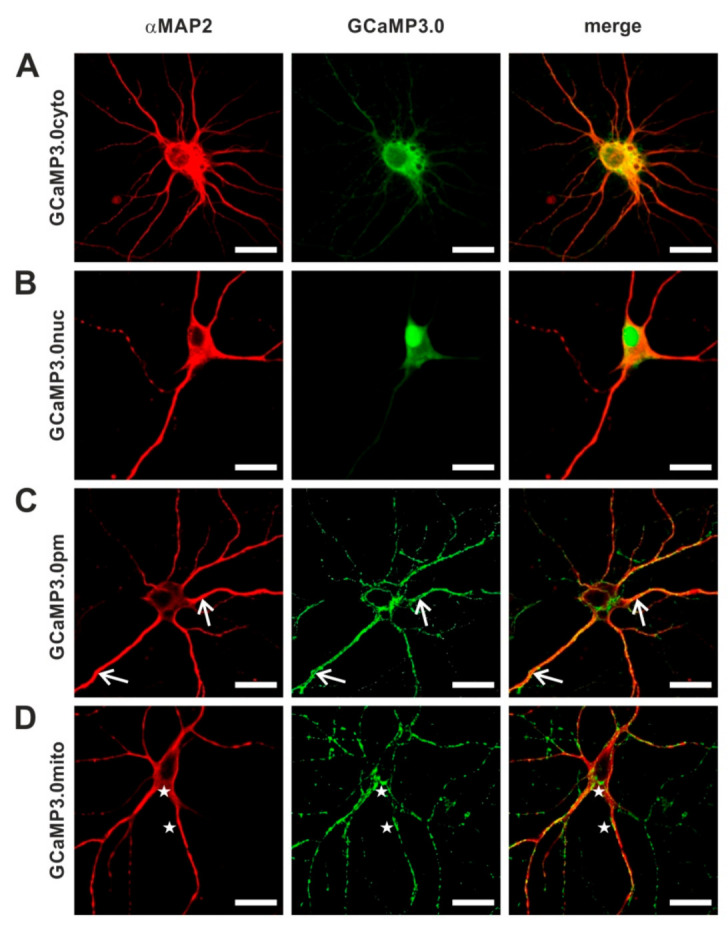
**Distribution of GCaMP3.0 variants in primary cortical neurons.** Rat embryonal cortical neurons were incubated for eight days with recombinant Adeno-associated virus preparations: (**A**) rAAV6-GCaMP3.0cyto, (**B**) rAAV6-GCaMP3.0nuc, (**C**) rAAV6-GCaMP3.0pm, and (**D**) rAAV6-GCaMP3.0mito. Samples were fixed (DIV16) and immunologically stained. Neurons were labeled with specific primary anti-MAP2 antibodies (rabbit-anti-MAP2, dilution 1:500) and secondary donkey anti-rabbit Cy3 antibodies (dilution 1:500; red). GCaMP3.0 variants were detected by their green fluorescence. Composite images are depicted on the right (merge). Subcellular localization of GCaMP3.0pm and GCaMP3.0mito is depicted with arrows and asterisks, respectively. The scale bar denotes 20 µm.

Since Ca^2+^ ions are intimately linked to neuronal signaling, we examined the suitability of all targeted GCaMP3.0 variants to report Ca^2+^ signals in primary cortical neurons. To deliver and express the sensors in primary neuronal cultures, we generated recombinant Adeno-associated viruses (rAAVs). In a previous study [57], we had uncovered that AAVs of serotype 6 efficiently transduced cortical neurons. Expression of GCaMP3.0 variants was examined by confocal fluorescence microscopy (Figure 8). Neurons were identified by immunological staining with specific α-MAP2 antibodies. Similar to the results obtained in stably transfected HEK293 cells, the targeted sensors were detected at their expected loci. The cytosolic version was predominantly found in the somatic cytosol but also in neurites (Figure 8A). The nuclear version was clearly enriched in the nucleus with small amounts of protein remaining in the somatic cytosol (Figure 8B). A clear and specific localization at the soma’s periphery as well as to the outer borders of neurites was observed for GCaMP3.0pm (Figure 8C, arrows).

Expression of GCaMP3.0mito resulted in a punctate distribution both at the cell’s soma and in neurites (Figure 8D, asterisks). The appearance was highly reminiscent of the distribution of mitochondria stained with MitoTracker [58].

**Figure 9:**
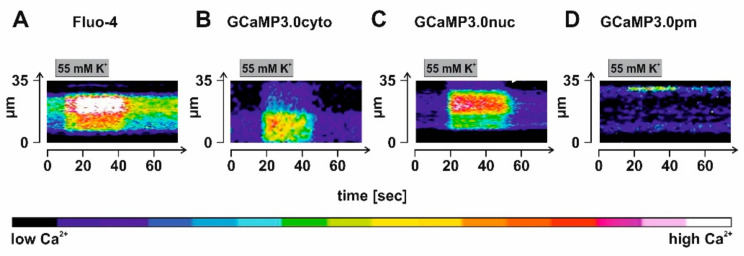
**Detection of depolarization-evoked Ca^2+^ signals in cortical neurons expressing GCaMP3.0 variants.** Rat embryonal cortical neurons were incubated for eight days with recombinant Adeno-associated virus preparations: (**B**) rAAV6-GCaMP3.0cyto, (**C**) rAAV6-GCaMP3.0nuc, and (**D**) rAAV6-GCaMP3.0pm. For control, samples were also loaded with the Ca^2+^-sensitive dye Fluo-4 (**A**). Depolarization of the membrane potential was achieved by superfusing the sample with a test solution containing a high K^+^ concentration (55 mM) for 30 s. Depolarization-evoked Ca^2+^ signals were registered in individual cells. A region of interest (ROI) along a line of 35 µm length was defined and the fluorescence intensity for each pixel along the line was extracted and color-coded. The analysis was repeated 75 times for each image of the stimulation series, and it is depicted as a time course.

To evoke Ca^2+^ signals, we incubated the samples in high K^+^ (55 mM) to depolarize the membrane potential. In Figure 9, results from representative experiments are shown. For data analysis, a ROI was defined given by a line of 35 µm length that was manually positioned in an image of a Fluo-4 loaded or rAAV-transduced neuron’s soma and for which the change in fluorescence had been registered. Fluorescence intensity of each pixel along the line was extracted, color coded, and then displayed against the time. Analysis was repeated on 75 individual images for each neuron. Stimulation of the samples was by superfusing a solution containing 55 mM K^+^ for 30 s onto the cells. Due to the geometry of the application stage, a delay of several seconds (4–5 s) occurred until the stimulus reached the cells. Nevertheless, the regime led to increasing fluorescent signals in all samples (Figure 9A–D). Notably, a highly localized change in fluorescence was observed in GCaMP3.0pm expressing neurons, supporting our previous notion that the sensors are specifically targeted to their predestined sub-cellular loci. Under these experimental conditions and considering the delay before the stimulus reached the samples, signal onset was in the range of one to a few seconds.

In summary, our data show that GCaMP3.0 variants were successfully expressed in primary cortical neurons where they reliably traced Ca^2+^ signals induced by membrane depolarization with pronounced spatial resolution.

## 3. Discussion

Here, we have generated and characterized subcellular targeted versions of the genetically encoded Ca^2+^ indicator (GECI) GCaMP3.0 for their ability in detecting changes in [Ca^2+^]_i_, both in cell-based pharmacological assays as well as in individual cells and neurons. Basic biophysical and biochemical properties of the variants were determined on bacterially expressed and affinity purified proteins.

A variety of sensors have been developed to register and to quantify dynamic changes of intracellular signaling molecules such as ions, small organic compounds, pH, or redox processes. Until this day, the families of genetically encoded indicators are continuously growing [5,29,59,60]. The concentration of cellular signaling molecules typically changes locally, necessitating that sensors report these dynamics in spatially restricted domains. To fulfill such requirements, sensors can be targeted to subcellular locations rather than being homogenously expressed, e.g., in the cytosol (for reviews see [59,60,61]). Since we were interested in studying the spatio-temporal dynamics of Ca^2+^ signals induced by, e.g., GPCR activation, we decided to employ GCaMP3.0 known as a robust Ca^2+^ detector with pronounced sensitivity and a dynamic range [34,62]. In addition to the cytosolic version of the protein, we generated GCaMP3.0 variants specifically targeted to subcellular loci. Targeting motifs had to be inserted 5′ to the M13 peptide-encoding sequence rather than to the 3′ end encoding the calmodulin module of the sensor because modifications at the C-terminus of the GECI either resulted in low expression rates, mis-localization, and/or loss of functionality. Most likely unrestricted conformational movement(s) or the general flexibility of calmodulin in GCaMP3.0 is required for its proper function.

For localization of GCaMP3.0 in the nucleus, a nuclear localization peptide originally identified in the SV40 large T antigen [51] was used. As expected, the modified protein (GCaMP3.0nuc) was preferentially expressed in the nucleus. Eventually due to constitutive expression in the stably transfected cell line, some GCaMP3.0nuc was present in the cytosol, which may limit analyses specifically addressing Ca^2+^ dynamics in the nucleus. Versions were also designed either to localize on the outer mitochondrial membrane (GCaMP3.0mom) or in the mitochondrial matrix (GCaMP3.0mito). Introducing the transmembrane anchor (TMA) derived from the proto-oncogene Bcl-2 [63] or a short sequence from the transport protein TOM20 [50] should guide GCaMP3.0 to the mitochondrial outer membrane. Both variants localized to subcellular organelles but only the TOM20-modified construct displayed co-localization with Mito Tracker Deep red, a dye specifically labeling mitochondria (Figure 1). Interestingly, the Bcl-2 modified protein displayed a less sharp and partly punctate staining. The staining pattern could be allocated to the Golgi apparatus. In cells co-transfected with pTagRFP-Golgi (Evrogen; [64]), which encodes a red fluorescent protein labeling the Golgi apparatus, fluorescent signals were co-localized (Figure S3). However, the Bcl-2 modified version was not further investigated here, whereas the TOM20-modified construct was, and it was named GCaMP3.0mom. In order to place GCaMP3.0 in the mitochondrial matrix, the initial 38 amino acid residues of cytochrome oxidase VIII (Cox8 motif) [48,49] were fused to the N-terminus of the GECI. The staining pattern was similar to that observed in cells transfected with GCaMP3.0mom. Transfecting cells with Cox8-modified GCaMP3.0 and counter staining with Mito Tracker Deep red finally proved localization of the GECI in mitochondria (GCaMP3.0mito; Figure 1(A4,D4,E4)). Since introducing targeting sequences to the N-terminus of GCaMP3.0 was successful so far, we used the same strategy to generate plasma membrane bound variants. The membrane localization sequence of the GTPase HRas (CAAX motif) [65] and the initial 21 amino acid residues of neuromodulin (Growth Associated Protein 43, GAP43) [45,46] were used for modification. It turned out that both versions were not anchored to the plasma when the targeting sequence was immediately fused to the initiating methionine of GCaMP3.0. In order to overcome possible masking effects of the M13 peptide when too close to the cell’s membrane, linker peptides (10 or 28 residues long) were inserted between the targeting sequence and the M13 peptide. Finally, the GAP43 peptide extended by a linker consisting of 28 residues resulted in preferential localization of GCaMP3.0 (GCaMP3.0pm) in the plasma membrane.

Previous studies have already delineated the influence of the spacing between cpEGFP and its flanking modules [66,67] in single fluorescent protein-based Ca^2+^ sensors. As we had also observed some impact of the targeting element’s position relative to the M13 peptide module, we decided to examine basic biophysical and biochemical properties of the modified GCaMP3.0 constructs in vitro. The variants were cloned into a bacterial expression vector allowing expression of His-tagged fusion proteins that were affinity purified. Using Ca^2+^ titrations, EC_50_ values for Ca^2+^ binding and the dynamic range of the proteins were assessed (Table 1). In comparison to the cytosolic version (GCaMP3.0cyto), neither the Ca^2+^ affinity nor the dynamic range of the modified sensors had changed, with the exception of a modest decrease for GCaMP3.0mito at a higher pH (see Table 1). This finding supported the rationale that these GCaMP3.0 variants could be used for measuring intracellular Ca^2+^ dynamics. However, performing in vitro titrations at different pH values, resulted in accelerated saturation of the sensor at pH 8.0 compared to pH 7.2 or 7.6. The influence of pH on the biophysical properties of a variety of GCaMP versions has been described previously [28,67], and it can be partly explained by the protonation status of the cpEGFP. Thus, when conducting time resolved measurements, one should consider that maximal ∆F values eventually could be reached faster in organelles differing in pH from the cytosol like in mitochondria, synaptic vesicles, or lysosomes. Because calibration of a sensor is typically performed using ‘cytosolic’ conditions, measurements should be performed mimicking the physiological conditions of the targeted organelle in advance.

One goal of our efforts to equip cells with GECI’s was to study [Ca^2+^]_i_ fluctuations induced by GPCR signaling. As a proof of principle, concentration-dependent responses of GCaMP3.0-transfected cell lines to histamine applications were measured. With EC_50_ values in the low micromolar range (1.1–2.7 µM), data obtained on Fluo-4 loaded, GCaMP3.0cyto- and GCaMP3.0nuc-expressing HEK293 cells were very similar. Thus, cells equipped with different GCaMP3.0 variants are compatible with high-throughput screening assays as employed in functional pharmacologic testing [68].

Histamine-evoked Ca^2+^ responses were also examined at the single cell level. Some cells showed oscillations of [Ca^2+^]_i_ as long as the ligand was present. Other cells showed single transient responses or oscillations on top of elevated Ca^2+^ levels. These response repertoires were reminiscent of GPCR-induced signaling reported previously [54,55] and most likely originated from cycles of GPCR de-sensitization and re-sensitization. However, histamine-evoked responses in this heterologous cell system were rather slow (seconds to tens of seconds) compared to Ca^2+^ transients occurring in neurons or glia cells [69,70] for which certain GCaMP variants have been developed and devoted to register fast kinetics of signaling molecules [71,72,73,74]. Notably, the GCaMP3.0 versions generated in this study were sufficiently sensitive and responded fast enough to follow the histamine-induced cellular Ca^2+^ signals described above.

Since Ca^2+^ ions play a pivotal role in neuronal signaling, especially in inter-neuronal communication mediated by neurotransmission, we also examined the functionality of the GCaMP3.0 variants in primary cortical neurons. To deliver and to express the sensors in primary neurons, we used rAAVs. In a previous study [57], we showed that rAAVs of serotype 6 efficiently transduced these neurons. Expression of the constructs was driven by a CaM Kinase II (CKII) promoter that is preferentially active in a broad spectrum of neurons. As for the transfected HEK293 cells, the GCaMP3.0 variants showed specific localization to subcellular structures. To induce Ca^2+^ signals, a high concentration of K^+^ (55 mM) was superfused onto the samples to depolarize the membrane potential. All variants tested reliably registered Ca^2+^ signals, which most likely originated from Ca^2+^ entry via voltage-dependent ion channels located in the plasma membrane. As already mentioned, the GCaMP3.0 sensor is less sensitive and slower in reporting changes in [Ca^2+^]_i_ than, e.g., GCaMP6 or GCaMP7 versions that have been developed for detecting even single Ca^2+^ sparks in neurons [4,7,72,73]. However, the specifically modified versions of GCaMP3.0 presented here extend and enrich the tool box of GECIs that can be used for functional measurements ranging from multi cell-based pharmacological screening assays to studying individual cell or neuron signaling behavior in response to external stimuli, with good temporal and spatial resolution.

## 4. Materials and Methods

### 4.1. Construction of Expression Vectors Encoding GCaMP3.0 Variants

We used a PCR-based modification strategy to introduce sequence motifs directing GCaMP3.0 to specific subcellular compartments. Reactions were performed on pCMV-GCaMP3.0 (kindly provided by Dr. L.L. Looger, Howard Hughes Medical Institute, Janelia Farm Research Campus, Ashburn, VA 20147, USA). Briefly, modifications were introduced at the 5′ end of the constructs with single to multiple rounds of overlapping PCRs. A restriction site (BamHI or XhoI) followed by a Kozak consensus motif (CCACC) [44] preceding the initiating ATG-codon of the open reading frame were introduced by the most distal 5′ primer. For all recombinants, the same 3′ primer was designed which harbored a stop codon followed by a BamHI restriction site. The 5′ initial nucleotide sequences of the different constructs are summarized in Table S1. Reactions were performed under standard conditions with: initial denaturation 94 °C, 2 min; followed by 30–35 cycles with denaturation 94 °C, 30 s, annealing at the appropriate melting temperature of the primer pair, 30 s, elongation 72 °C, 45 s with KOD Hot Start DNA polymerase (Merck, Darmstadt, Germany). For expression in eukaryotic cells, fragments were subcloned into pcDNA6/myc-His A (Life Technologies/Thermo Fisher Scientific, Darmstadt, Germany) or pscCMV/pscCKII, a vector used for production of recombinant Adeno-associated viruses (rAAVs; kindly provided by Dr. H. Büning, MHH Hannover, Germany). All constructs were sequence-verified (MWG/Operon, Ebersberg, Germany). The following variants (ordered according to their subcellular localization) were obtained: GCaMP3.0cyto (cytoplasmic), GCaMP3.0mito (mitochondrial matrix), GCaMP3.0nuc (nucleus), GCaMP3.0pm (plasma membrane), GCaMP3.0mom (mitochondrial outer membrane), and GCaMP3.0golgi (Golgi apparatus).

### 4.2. Generation of Stably Transfected HEK293 Cell Lines

Human embryonic kidney cells (HEK293; #85120602, ECACC, Porton Down, Salisbury, UK) were transfected with 10 µg of the different GCaMP3.0 constructs in pcDNA6/myc-His A by a modified calcium phosphate method [75] following a previously established protocol [76]. Transfected cells were selected in the presence of the antibiotic Blasticidin (0.01 mg/mL). Expression of GCaMP3.0 was monitored by its green fluorescence via fluorescence microscopy.

### 4.3. Production of rAAVs

Recombinant Adeno-associated virions (rAAV) were generated by transient transfection of HEK293 cells (#CRL-1573, ATCC, Teddington, Middlesex, UK). Cells were cultivated in DH10 medium (DMEM + Glutamax (Invitrogen/Thermo Fisher Scientific, Darmstadt, Germany), 10% (*v/v*) FBS (Gibco/Thermo Fisher Scientific, Darmstadt, Germany), 1% (*v/v*) antibiotics/antimycotics (Invitrogen/Thermo Fisher Scientific)) at 37 °C, 5% CO_2_ and 95% relative humidity. Approximately 2 × 10^7^ cells on Ø 14.5 cm dishes were triple-transfected with the vector plasmid providing the transgenic viral genome as well as the helper plasmids pRC [77] and pXX6-80 [78], using the calcium phosphate method (s.a.). After 24 h incubation at 37 °C, 5% CO_2_, and 95% relative humidity, the medium was exchanged for DH10 with reduced FBS (2% (*v/v*)). Cells were harvested in PBS-M/K (130 mM NaCl, 2.5 mM KCl, 1 mM MgCl_2_, 70 mM Na_2_HPO_4_, 30 mM NaH_2_PO_4_, pH 7.4) after 24 h and lyzed in 150 mM NaCl, 50 mM Tris/HCl, pH 8.5 by four freeze/thaw-cycles in liquid nitrogen and at 37 °C. Nucleic acids were digested with benzonase (50 U/mL; Merck) for 30 min at 37 °C. After removal of cell debris by low-speed centrifugation, rAAV particles were enriched by density gradient centrifugation. The rAAV suspension was sub-layered with iodixanol (Sigma-Aldrich, Taufkirchen, Germany) solutions (15%, 25%, 40%, and 60% iodixanol) and centrifuged (264,000 *g*, 4 °C, 2 h). The 40% iodixanol phase containing virus particles was collected and viral titers were determined by quantitative PCR applying a primer pair binding to the CaMKII promoter (5′ primer GCAAAGAGGAGCAGGTTTTG, 3′ primer CTGAGTGCAAACGGAGAACC).

### 4.4. Expression and Purification of GCaMP3.0 Variants from E. coli Cells

For overexpression of GCaMP3.0 proteins, the *E. coli* strain BL21(DE3)-pLysS was used. Sensor-encoding DNA fragments were subcloned into pRSET A expression vector (Invitrogen/Thermo Fisher Scientific). This strategy led to adding a hexa-histidine (His_6_) tag to the N-terminus of each construct facilitating affinity purification via Nickel-nitrilotriacetic acid (Ni-NTA; Macherey and Nagel, Dueren, Germany) agarose matrix. Expression of sensor proteins was induced with 1.0 mM Isopropyl-β-D-thiogalactopyranosid (IPTG) at 20 °C for 18 h. Purification of proteins by Ni-NTA chromatography was performed according to the supplier’s protocol.

### 4.5. Spectroscopic Characterization of Purified GCaMP3.0 Variants

To examine fluorescent properties of the modified GCaMP3.0 sensors, emission spectra at different Ca^2+^ concentrations ([Ca^2+^]_free_) were registered. Purified protein was diluted in Ca^2+^ free buffer (30 mM 3-(N-Morpholino)propansulfonic acid (MOPS), 100 mM KCl, 10 mM K_2_EGTA, pH 7.2) to a final concentration of 2 µM. By adding a Ca^2+^-containing buffer (30 mM MOPS, 100 mM KCl, 10 mM CaEGTA, pH 7.2) the [Ca^2+^]_free_ was successively increased. Values of [Ca^2+^]_free_ were calculated with WEBMAXC (Maxchelator, http://www.stanford.edu/~cpatton/maxc.html (accessed on 18 March 2016)). Samples were excited at 470 nm and emission spectra were recorded between 480 and 600 nm with a QM-4 spectrometer (PTI; Horiba Scientific, Piscataway, NJ 08854, USA). Data analysis was performed with Graph Pad Prism 5.04 (GraphPad; San Diego, CA 92108, USA). Determination of GCaMP3.0 fluorescence at pH 7.6 and 8.0 was according to a protocol established by [79].

### 4.6. Ca^2+^ Fluorimetry in Stably Transfected Cell Lines and Primary Cortical Neurons

To monitor changes of [Ca^2+^]_i_ in single cells, two-photon-laser scanning fluorescence microscopy measurements were performed. Details of the setup are described in [55]. Cells were grown on poly-L-lysine (PLL; 0.1 mg/mL) coated coverslips in 24 well plates. Coverslips were transferred to a measuring chamber connected to a perfusion system that allowed applying test solutions by gravital flow. Changes in [Ca^2+^]_i_ were registered either in Fluo-4 or Fura-2 loaded cells or in cells expressing GCaMP3.0 variants. Loading with Fluo-4 was in extracellular solution (ES; 120 mM NaCl, 5 mM KCl, 2 mM MgCl_2_, 2 mM CaCl_2_, 10 mM HEPES, 10 mM Glucose, pH 7.4 (NaOH)) supplemented with 1.7 µM Fluo-4 AM or 2 µM Fura-2 AM (both from Molecular Probes/Invitrogen/Thermo Fisher Scientific, Darmstadt, Germany), 3 mM probenecid, and 0.02% (*w/v*) pluronic F-127 (Sigma-Aldrich) for 30 min at room temperature. For stimulation of endogenous Ca^2+^ signaling cascades, cells were superfused with ES solution containing different histamine concentrations or high K^+^ (55 mM). Two-photon excitation of samples was at 760 nm (Fura-2), 800 nm (Fluo-4), or 920 nm (GCaMP3.0). Fluorescence emission was registered at wavelengths > 500 nm. For each sample 70 to 800 images were registered with scanning speeds between 0.5–1 images/sec. Images contained 256 × 256 to 512 × 512 pixels. Data were analyzed with Image J (https://imagej.nih.gov/ij/ (accessed on 21 March 2011), 1997–2018; National Institute of Health, Bethesda, Maryland, USA). A region of interest (ROI) was defined. The mean fluorescence intensity was calculated for all individual images of a sample and plotted versus time (=duration of the experiment). Using the maximal change in fluorescence evoked by the different ligand concentrations, concentration–response curves (∆F vs. ligand concentration) were calculated using Graph Pad Prism 5.04.

### 4.7. Preparation of Primary Cortical Neurons and rAAV Transduction

Primary cortical cultures were prepared as previously described [57,80]. Briefly, cortices from embryonic day 18 (E18) Wistar rat (Charles River, Sulzfeld, Germany) brains were dissected and mechanically dissociated by trituration with a fire-polished, salinized Pasteur pipette in 1 mL Hank’s balanced salt solution without calcium or magnesium (HBSS-) (0.035% sodium bicarbonate, 1 mM pyruvate, 10 mM HEPES, 20 mM glucose, pH 7.4). The cell suspension was diluted 1:2 in HBSS+ (=HBSS with calcium and magnesium) and non-dispersed tissue was allowed to settle for 3 min. The supernatant was centrifuged for 2 min at 200 g. The pellet was resuspended in a 1 mL Neurobasal medium (Gibco, Paisley, UK) supplemented with 1% B-27 (Gibco, Grand Island, NY, USA), 0.5 mM L-glutamine (Gibco), and 50 µg/mL gentamicin (Sigma-Aldrich) per hemisphere. Cells were plated in a concentration of 50,000 cells per well (24-well-plate) on poly-L-lysine (PLL; 10 µg/mL) coated 12 mm coverslips. Cells were kept at 37 °C, 5% CO_2_, and 95% humidity in a 500 µL medium. Primary cortical cells were transduced with rAAVs on day in vitro (DIV) DIV8 with different multiplicities of infection (MOI). The medium (500 µL) was completely exchanged with medium containing 10^7^, 10^8^, 10^9^, 10^10^, or 10^11^ viral genomes to achieve MOIs of 2·10^2^, 2·10^3^, 2·10^4^, 2·10^5^, and 2·10^6^ viral particles per cell (vp/cell), respectively. As a control, the same volume of 40% Iodixanol was added to individual wells. Three days post transduction (DPT), 100 µL medium was added. Cells were assessed for functional measurements or fixed for immunostaining on DIV14.

### 4.8. Immunological Staining of Cell Lines and Primary Cortical Neurons

Cells used for immunostaining were grown on cover slips, fixed with 4% (*w/v*) paraformaldehyde in PBS (PFA) at RT for 20 min and washed with 1x PBS (pH 7.4). Unspecific binding sites in HEK293 cells were blocked for 30 min in a phosphate buffer containing 0.5% (*w/v*) Triton-X100 and 5% (*w/v*) chemiblocker (Merck, Darmstadt, Germany). Unspecific binding sites in neurons were blocked overnight in PBS containing 1% (*w/v*) BSA and 2% (*v/v*) heat inactivated goat serum. The following primary antibodies were used: rabbit (rb)-anti-calnexin (dilution 1:100; #2674, Cell Signaling Technology, Danvers, MA 01923, USA) and (rb)-anti-MAP2 (dilution 1:500; #AB5622, Chemicon/Millipore, Darmstadt, Germany). Primary antibodies were visualized using donkey (dk)-anti-rbCy3 (dilution 1:500; #711-166-152, Dianova, Hamburg, Germany) and dk-anti-rbAlexa(A)594 (dilution 1:1000; #711-586-152, Dianova) fluorescently-labeled secondary antibodies in blocking solution. Nuclei were stained with TOPRO-3 (dilution 1:500 in blocking solution; Life Technologies/Thermo Fisher Scientific). Mitochondria were stained with 100 nM MitoTracker (Deep Red FM; Life Technologies/Thermo Fisher Scientific) for 20–30 min at 37 °C, 5% CO_2_, and 95% humidity immediately before fixation of the sample with 4% PFA. Samples were mounted in Aqua-Poly/Mount (Polysciences, Eppelheim, Germany) and examined using a Confocal laser scanning microscope (Leica TCS SP5; Leica Microsystems, Heidelberg, Germany).

## Figures and Tables

**Table 1:** Mean values for half-maximal stimulation (EC_50_ [nM]) and dynamic range of GCaMP3.0 variants by [Ca^2+^]_free_. Values were obtained from non-linear fitting of the data (*n* = number of experiments) from concentration–response curves (GraphPad Prism 5.04).

Protein	*n*	EC_50_ [nM]	Dynamic Range
GCaMP3.0cyto	10	234 ± 28	9 ± 2
GCaMP3.0pm	6	261 ± 63	10 ± 1
GCaMP3.0mito (pH7.2)	5	264 ± 13	10 ± 2
GCaMP3.0mito (pH7.6)	5	326 ± 31	6.5 ± 1
GCaMP3.0mito (pH8.0)	5	151 ± 30	4.8 ± 0.1
GCaMP3.0mom	4	294 ± 41	11 ± 1
GCaMP3.0nuc	4	286 ± 13	11 ± 1

## Data Availability

Data and materials described in this study are available upon request from the corresponding author.

## Supplementary Materials

The supporting information can be downloaded at: https://www.mdpi.com/article/10.3390/ijms23126593/s1.
