# Supplementary material for: The Functional Characterization of GCaMP3.0 Variants Specifically Targeted to Subcellular Domains
Source: Int J Mol Sci. 2022 Jun 13;23(12):6593. doi: 10.3390/ijms23126593 (PMC9223625; doi:10.3390/ijms23126593)
Supplement: Supplementary file 1 [file ijms-23-06593-s001.zip › ijms-1742187-supplementary.pdf]

Supplementary Materials

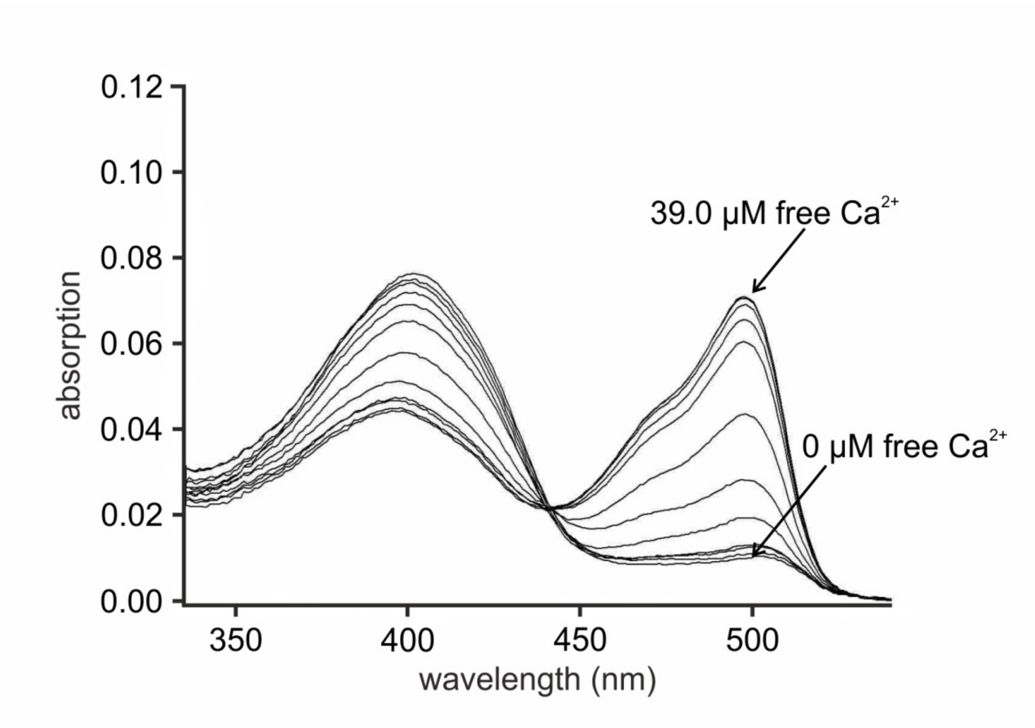

Figure S1. In vitro characterization of GCaMP3.0nuc. The construct was cloned into pRSET A vector, over-expressed in E. coli (BL21(DE3)LysS cells and affinity purified via Ni-NTA agarose. Purified GCaMP3.0nuc protein (2 µM final concentration) was diluted in incubation buffer (30 mM MOPS, 100 mM KCl, pH 7.2) adjusted to the desired $[Ca^{2+}]_{free}$ with CaEGTA (10 mM)/K_2EGTA (10 mM). For each $[Ca^{2+}]_{free}$ the absorption spectrum was registered. Spectra obtained at 0 µM and 39.0 µM $[Ca^{2+}]_{free}$ are indicated with arrows.

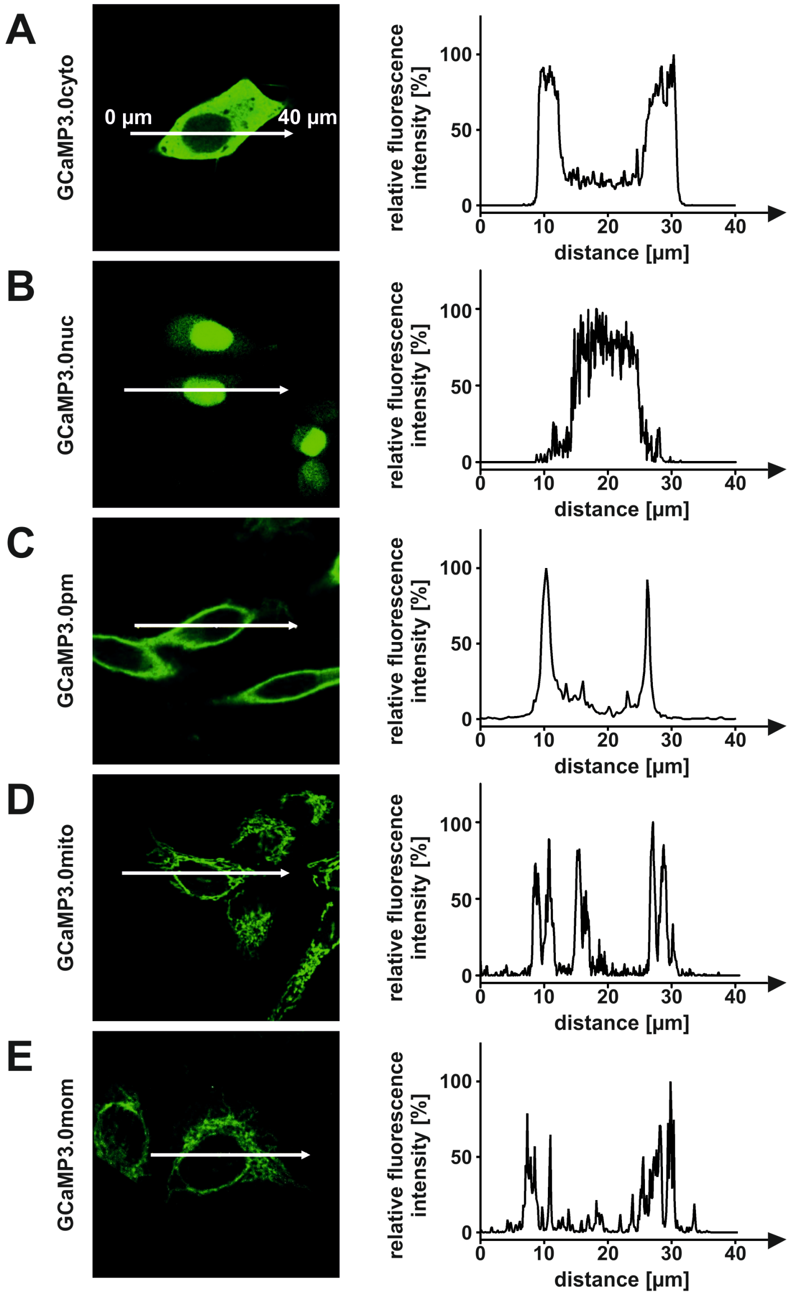

Figure S2. Cellular distribution of GCaMP3.0 variants. Sub-cellular localization of GCaMP3.0 variants was determined in individual cells of stably transfected cell lines. Human embryonic kidney (HEK293) cells were used for transfection. Line scan analyses were performed. A region of interest (ROI) was defined along a line (white arrow) in confocal fluorescence images of individual cells (left panel). The line had a total length of 50 µm. The first and the last 10 µm were placed outside of the cell. Relative fluorescence intensity along the line was plotted against the distance (right panel). Cell lines expressed (A) GCaMP3.0cyto, (B) GCaMP3.0nuc, (C) GCaMP3.0pm, (D) GCaMP3.0mito, or (E) GCaMP3.0mom.

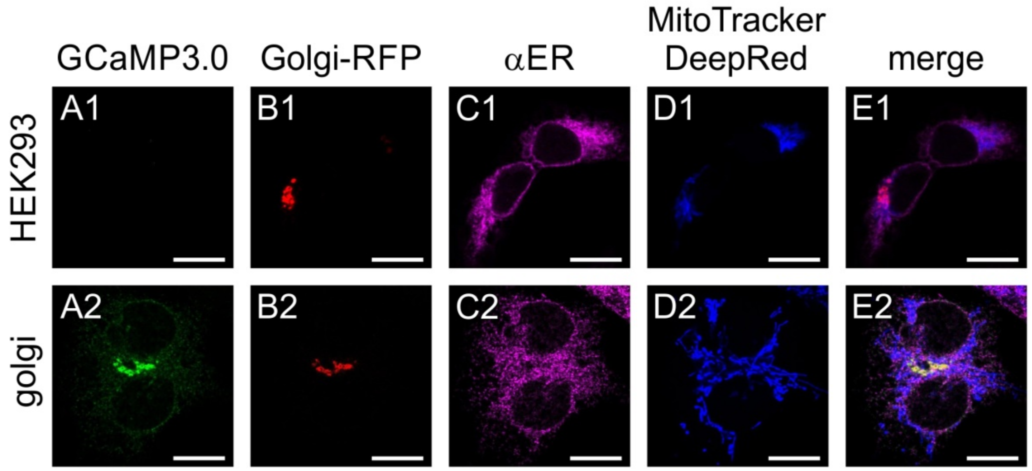

Figure S3. Cellular localization of Bcl-2 modified GCaMP3.0. HEK293 cells were either non-transfected with a GCaMP3.0 encoding construct (A1-E1) or with GCaMP3.0golgi (A2-E2). Note that panel A1-E1 is taken from Figure 1. Expression of GCaMP3.0golgi was detected by its green fluorescence (A2). For identification of the Golgi apparatus cells were transfected with Golgi-RFP (B1; B2). The endoplasmic reticulum was stained with primary rabbit anti-calnexin antibodies (dilution 1:100) and secondary donkey anti-rabbit-A594 (dilution 1:1000) antibodies (C1; C2). Labeling of mitochondria was performed with MitoTracker DeepRed FM (D1; D2). Fluorescent images were obtained by confocal imaging of the samples. Composite images are depicted in (E1; E2). The scale bar denotes 8μm.

Table S1. Initiating nucleotide sequences of GCaMP3.0-encoding constructs. In each construct, the 5′ restriction site, the Kozak consensus sequence (ccacc; italic) and the initiating ATG-codon (bold) of the open reading frame is given. Nucleotides encoding targeting peptides are highlighted in grey, the linker-encoding sequence in GCaMP3.0pm is highlighted in yellow, and the start of the GCaMP3.0 encoding sequence is highlighted in green.

**GCaMP3.0cyto:**
tctaga*ccacc***ATG**ATGGGTTCTCATC
**GCaMP3.0nuc:**
tctaga*ccacc***ATG**CCGAAGAAGAAGCGCAAGGTGATC**ATG**GGTTCTCATC
**GCaMP3.0pm 28aa-GAP43:**
ggat*ccacc***ATG**CTGTGCTGTATGAGAAGAACCAAACAGGTTGAAAAGAATGATGAGGACAAAAGATC GATGACTGGATGGAGAACATTGACGTGTGTGAGAACTGCCATTATCCCC CGCCGAAGCTTCGAAT TGATCCACC GGATCTCGCCACC**ATG**GGTTCTCATC
**GCaMP3.0mito:**
tctaga*ccacc***ATG**TCCGTCCTGACGCCGCTGCTGCTGCGGGGCTTGACAGGCTCGGCCCGGCGGCTCCC AGTGCCGCGCGCCAAGATCCATTCGTTGGGCGATCCACCGGTCGCCACG**ATG**GGTTCTCATC
**GCaMP3.0mom:**
tctaga*ccacc***ATG**GTGGGTCGGAACAGCGCCATCGCCGCCGGTGTATGCGGGGCCCTTTTCATTGGGTA CTGCATCTACTTCGACCGCAAAAGACGAAGTGACCCCAAC**ATG**GGTTCTCATC
**GCaMP3.0golgi:**
tctagaccacc**ATG**AAGACGTTACTCTCCCTCGCCCTCGTCGGCGCCTGCATCACGCTCGGCGCCTACCT CGGCCATAAGATG**ATG**GGTTCTCATCATC
